# AhR pathway activation prevents food allergy in mice partly by preserving CD25-positive Tregs in the thymus

**DOI:** 10.1186/2045-7022-3-S3-P43

**Published:** 2013-07-25

**Authors:** R Pieters, V Schulz, M Bol-Schoenmakers, J Smit

**Affiliations:** 1 Institute for Risk Assessment Sciences, Utrecht University, Utrecht, the Netherlands

## Background

Food allergy is an increasing health problem. We and others have shown that the intensity of food allergic reactions can be regulated by regulatory T (T­­_reg_) cells. In addition, others have shown that activation of the aryl hydrocarbon receptor (AhR) is able to induce T_reg_ cells. Here, we investigated whether activation of the AhR could suppress food allergic responses through the induction of T_reg_ cells in a mouse peanut allergy model.

## Methods

C3H/HeOuJ mice were exposed to AhR ligands (TCDD, 6-formylindolo[3,2-b]carbazole (FICZ), β-naphthoflavone (β-NF) and 6-methyl-1,3,8-trichlorodibenzofuran (6-MCDF)) before and during sensitization to peanut. The latter was done by gavaging peanut extract (PE)+cholera toxin. Effects on antibody levels, mast cell responses and cytokine production was investigated. The role of CD4^+^CD25^+^Foxp3^+^ T_reg_ cells was investigated by depleting these cells with anti-CD25 mAb (ip) during sensitization to PE.

## Results

A dose of 15 µg/kg BW TCDD caused a decrease in levels of PE-specific IgE, IgG1 and IgG2a, mast cell degranulation (mMCP-1) and PE-induced IL-5, IL-10 and IL-13) and an increase in the percentage of CD4^+^CD25^+^Foxp3^+^ T_reg_ cells. The suppressive effect of AhR activation on the peanut allergic response was reversed in the absence of CD4^+^CD25^+^Foxp3^+^ T_reg_ cells. Careful identification of the thymus revealed that the increase percentage of CD4^+^CD25^+^Foxp3^+^ T_reg_ cells might result from selective survival of these cells from TCDD-induced thymus atrophy. None of the other ligands FICZ, β-NF and 6-MCDF, were effective in preventing sensitization to peanut, although NF may influence the levels of some cytokines (IL5, IL10, IFN-g, IL17a). Differences between TCDD and FICZ, β-NF and 6-MCDF may be explained by differences in binding affinity to the AhR and effectiveness to activate AhR-dependent gene transcription but also by differences in susceptibility to metabolic conversion.

## Conclusion

Together, activation of the AhR (by its high affinity ligand TCDD) during sensitization suppresses the peanut allergic response and CD4^+^CD25^+^Foxp3^+^ T_reg_ cells are involved in this suppression. The increase of these natural Tregs occurs in part because they survive thymolytic effects of TCDD. Data suggest that the AhR pathway may be relevant in modulating Th2 responses and possible target of therapeutic leads.

## Disclosure of interest

None declared.
